# Patterns of Prior Induced Abortions and the Likelihood of Subsequent Natural Pregnancy Loss: Exploratory Application of Pregnancy Outcome Sequencing

**DOI:** 10.2196/78489

**Published:** 2025-12-09

**Authors:** Francois K Domagni, Daniel J Tsang, James Studnicki, John W Fisher, David C Reardon, Christopher Craver

**Affiliations:** 1 California State University, Northridge Northridge, CA United States; 2 University of North Carolina at Charlotte Charlotte, NC United States; 3 Charlotte Lozier Institute Arlington, VA United States; 4 Elliot Institute Gulf Breeze, FL United States

**Keywords:** reproductive history, pregnancy outcomes, abortion, natural loss, miscarriage, birth

## Abstract

**Background:**

Research concerning the long-term health consequences of induced abortion is constrained by both the limitations in the availability of data necessary to construct complete reproductive histories, as well as the limitations in the analytical methods necessary to interpret them.

**Objective:**

This study aims to determine the association of induced abortion and the likelihood of a subsequent natural loss by applying the pregnancy outcome sequence (POS), a research construct that defines the number and order of all pregnancy outcomes (births, induced abortions, natural losses) in each woman’s reproductive history.

**Methods:**

Using the Medicaid Analytic eXtract files from the Centers for Medicare and Medicaid Services Chronic Conditions Warehouse, we identified a study population of 508 unique POSs, representing 5455 women, each of whom had 1 to 16 pregnancy outcomes, for a total of 14,198 pregnancies. We applied an exploratory iterative sequential analytical approach, which included aggregate POS correlation analysis, logistic multiple regression, and simultaneous CIs (Agresti and Tukey-Kramer methods). We also established counting methods to populate the data tables for each analytical phase.

**Results:**

Overall, we found evidence to conclude that both prior abortions and natural losses are significantly associated with the risk of subsequent natural losses. For abortion, there is evidence of a dose-response relationship from 0 to 3 abortions and the likelihood of subsequent natural loss. For natural loss, the risk of a subsequent natural loss is significant after 2 natural losses and between the first and second. There is no association of prior births, or any combination of birth/abortion or birth/natural loss, with the risk of subsequent natural loss. Limitations imposed by the skewed distribution of the number of total pregnancies per reproductive history, resulting in small cell sizes, and exclusion of important covariates, restrain assurance in the results.

**Conclusions:**

The POS demonstrates that the order and combinations of pregnancy outcomes may result in varying conclusions that were previously undetectable. Therefore, further methodological development is indicated.

## Introduction

### The Knowledge Gap: Toward a Comprehensive Interpretation of Reproductive History

A pregnancy can end in one of 3 ways: an induced abortion, a live birth, or a natural loss. The terms spontaneous abortion, miscarriage, and early natural pregnancy loss are often used interchangeably. In studies of various domains of reproduction, the pregnancy outcomes are considered as both dependent and independent variables. In the former case, the pregnancy outcome is seen as the result of a factor or group of factors that influence the likelihood that the outcome will occur; for example, chromosomal abnormalities and miscarriage, and poverty and abortion [1,2]. In the latter case, the pregnancy outcome is perceived as a precipitating or causative factor for some other outcome; for example, abortion and subsequent repeat abortions, and miscarriage and anxiety and depression [3,4]. Most of these studies are limited by the data available to a single pregnancy outcome as the focus of the analysis, such as the woman’s first pregnancy or another identified “index” outcome. However, for most women, a single pregnancy outcome fails to capture the full extent of variation in their reproductive years.

The number, type, timing, and order of all pregnancy outcomes comprise an important component of a woman’s reproductive history. This is illustrated by conflicting research on the association between induced abortion and subsequent pregnancy loss. The science related to the various influences and associations of induced abortion to other outcomes is a more contemporary component of the concept of a woman’s “reproductive history,” a term used by researchers for generations and introduced as a PubMed search term in 1997 [5]. Reproductive history has been defined as “An important aggregate factor in epidemiological studies of women’s health. The concept usually includes the number and timing of pregnancies and their outcomes, the incidence of breastfeeding, and may include age of menarche and menopause, regularity of menstruation, fertility, gynecological or obstetric problems, or contraceptive usage” [6]. Research on the association between abortion and subsequent natural loss has produced conflicting results [7-14]. A major methodological limitation confronting analyses of pregnancy outcomes is the reliance on patient recall rather than the objective documentation of one or more abortions. Since randomized controlled trials are not appropriate for studying the risks of abortion, investigators usually use observational study designs (eg, cross-sectional studies or cohorts), and use some form of survey presenting the possibility of both recall and selection bias [15]. Women with abortions tend to underreport having had an abortion when interviewed or responding to a questionnaire [16]. An important finding concluded that women actually identify less than half (47%) of the abortions they experienced [17]. The recall bias may also be selective; that is, it has been demonstrated that healthy participants with an exposure (abortion) are less likely to report their abortion if they have not experienced long-term negative health effects [18]. Actual selection bias occurs when some factor or variable is associated with the outcome of interest. Investigators now routinely include levels of postpregnancy outcomes in the period prior to the pregnancy as a means to control selection bias [19]. Of course, recall bias and other problems associated with survey data are best addressed by using data extracted from a comprehensive registry or medical record summary, which, if complete, provides an accurate record of all pregnancy outcomes within a reproductive history.

We consider the pregnancy outcome sequence (POS) as an analytical construct. A research construct represents an attempt to represent some abstract phenomenon that is not directly measurable. For example, “intelligence” (which is not directly measurable itself) is inferred from other variables that are measurable and observable, such as scores on IQ tests, formal educational attainment, and problem-solving skills. These observable variables are selected to validate the underlying concept represented by the construct; that is, they operationalize it. The POS construct must be operationalized to capture or validate the concept it represents and may have many measurable variables; that is, the total number of outcomes in the POS; the first pregnancy outcome; the time interval between the first and last pregnancy outcome; the percentage of all outcomes represented by a particular outcome; and so on. The POS construct may then enable an explanation or understanding of complex relationships that are hidden in the patterns among the variables that compose the construct.

However, the ordering of pregnancy outcomes is exponentially complex; that is, the number of unique combinations possible increases more and more with each additional pregnancy. A woman with 4 pregnancies during her reproductive years will have 81 different possible orderings of these pregnancy outcomes, 6561 if she has 8 pregnancies, and more than 43 million if she is pregnant 16 times. Methods which attempt to describe the characteristics of this complexity and also interpret its influence on or association with other factors or events are largely absent from the research domain. The availability of and access to data sources necessary to inform these analyses are similarly limited and restricted. This exploratory research attempts an explanation of the likelihood of natural loss given the number, type, and patterns of the order of all pregnancy outcomes.

### Benefits of Sequential Analysis

Longitudinal data is typically composed of sets of categorical sequences. Sequential data is arranged in sequences where the order matters. In a period of hospitalization, for example, the medical record is a repository of tests, procedures, and other treatment events, of which patterns emerge with analysis. In the typical descriptive ordering analysis of the association of a particular pregnancy outcome (abortion) with the likelihood of another (miscarriage), the outcome that was closest in time to the subsequent outcome is used for classifying the type of outcome and determining the interpregnancy interval [20]. In sequential analysis, incremental findings may allow a clearer understanding of complex relationships, which may also enable investigators to be more flexible during the research project. In this analysis, for example, a consistent lack of association between birth and subsequent natural loss enabled investigators to pursue a set of strategies to increase sample size. In sum, determining within-sequence patterns for every pair of pregnancy outcomes in every reproductive history may reveal associations among outcomes that were undetectable without this level of outcome granularity and order.

In this exploratory analysis, we introduce the POS in order to meet 3 analytical objectives. First, we will comprehensively describe the POSs that are extant in a defined population. To our best knowledge, such a description has never been previously accomplished. Second, we will provide a preliminary assessment of the applicability and utility of the POS as a research construct within this research domain. What are the challenges and apparent inadequacies in this application? What are the next steps and possible future applications? Third, we will determine whether this initial analysis supports, refutes, or is inconclusive regarding the association of patterns of abortion and the likelihood of subsequent natural pregnancy loss. Given the exploratory and developmental aspects of this project, no finding would be considered confirmatory or conclusive.

## Methods

### Data Source and Study Population Coding

This study used data drawn from the Medicaid Analytic eXtract files licensed through the Centers for Medicare and Medicaid Services Chronic Condition Data Warehouse. The total dataset comprises Medicaid-eligible women 13 years of age and older, with at least one pregnancy, from 17 states where state funds provided coverage for induced abortions ineligible for coverage by federal Medicaid. Medicaid eligibility is determined by financial status relative to a designated poverty level, and, therefore, the study results may not be generalizable to more affluent women. In this analysis, the study population was limited to 7 states that continually submitted their full Medicaid claims data for all years of the study period (1999-2015): Connecticut, New Jersey, New Mexico, New York, Oregon, Vermont, and West Virginia. Additionally, to be included in the study population, each patient had to maintain Medicaid eligibility for at least one month in each of the 17 years (1999-2015). This criterion was implemented to prevent pregnancies from being missed due to nonreporting by the states or interruptions in patient eligibility. Study participants were drawn from all women who were or turned 16 years old in 1999. In a similarly determined population of 15-year-old women in 1999, less than 0.47% had a pregnancy, providing high confidence that this study reflects the outcomes of women’s first and subsequent pregnancies. These inclusion criteria also provide a control on the range of women’s age at the pregnancy outcome since women are all less than 33 years of age during the observation period, largely avoiding the increasing risk of natural loss in later years [21]. We identified all unique pregnancy outcomes using *ICD-9* (*International Classification of Diseases, Ninth Revision*) and *ICD-10* (*International Statistical Classification of Diseases, Tenth Revision*) codes. Pregnancy outcomes were confirmed using Current Procedural Terminology, Fourth Edition (CPT4) and Healthcare Common Procedure Coding System codes.

The following codes were used to identify pregnancy outcomes in 3 categories: live birth (*ICD-9* V27.0, V27.2, V27.5 and *ICD-10* Z370, Z372, and V375); induced abortion (*ICD-9* 635.xx; *ICD-10* O04; CPT4: 59840, 59841, 59850, 59851, 59852, 59855, 59856, and 59857; and Healthcare Common Procedure Coding System: S0199, S2260, S2265, S2266, S2267, X7724, X7726, S0190, and S0191); and natural pregnancy loss, a category that includes miscarriage, stillbirth, ectopic pregnancy, and other pregnancies not ending in induced abortion or live birth (*ICD-9* V27.1, V27.4, V27.7, 630, 631, 633, and 634; ICD-10 O00, O01, O02, and O03). Multiple codes occurring within 30 days of an induced abortion or 180 days of a live birth were collapsed into a single pregnancy outcome based on the first date associated with that group of claims. Twins and higher-order pregnancies that resulted in a combination of live birth and pregnancy loss were excluded.

In the typical multivariate model application, the addition of important variables is encouraged, consistent with model parsimony; that is, simpler models are preferred over more complex ones, provided the models fit the data equally as well. This approach implies that key variables such as comorbid conditions, age at pregnancy outcome, race, type of abortion (medication vs surgical), type of natural loss (miscarriage, stillbirth, and ectopic and molar pregnancy), type of delivery (vaginal vs c-section) and others could influence the likelihood of the subsequent (ie, next pregnancy) outcome, and should therefore be included in the analysis. This research is atypical for multiple reasons. The distribution of the number of pregnancy outcomes within a population of reproductive histories is highly skewed, with nearly three-quarters of women experiencing 3 or fewer pregnancies. At the same time, the number of unique POSs increases exponentially for each additional pregnancy. From a research design perspective, this means that sample sizes become smaller and smaller as the number of possible unique sequences increases. This enables more possible combinations, which are rarely populated or are absent completely from the study population. Adding variables to the model in these circumstances aggravates the problems of statistically underpowered cell sizes and the instability of estimates. Therefore, we concluded that this first exploratory application of sequential analysis to pregnancy outcomes should be parsimonious so as to enable an iterative application of multiple analytical methods that reflect interim results [22].

### Ethical Considerations

This study has been exempted from institutional review board review by Sterling Institutional Review Board (IRB ID 7269).

### POS as a Research Construct

The POS is a numeric construct representing the occurrence of each of these possible outcomes in the order in which they occur, coding an abortion as a 1, a live birth as a 2, and a natural loss as a 3. For instance, if a woman has had a total of 7 pregnancies with an outcome sequence of birth-abortion-natural loss-birth-abortion-abortion-natural loss appearing in that order, her history would be coded as a POS 2-1-3-2-1-1-3. All women in the data set who experienced this same series of pregnancy outcomes in the same order were assigned this POS. The number of women with the same POS was recorded, and their age at each outcome event was averaged and included in the POS table.

### Counting Methods

#### POS Description

**Figure 1:**
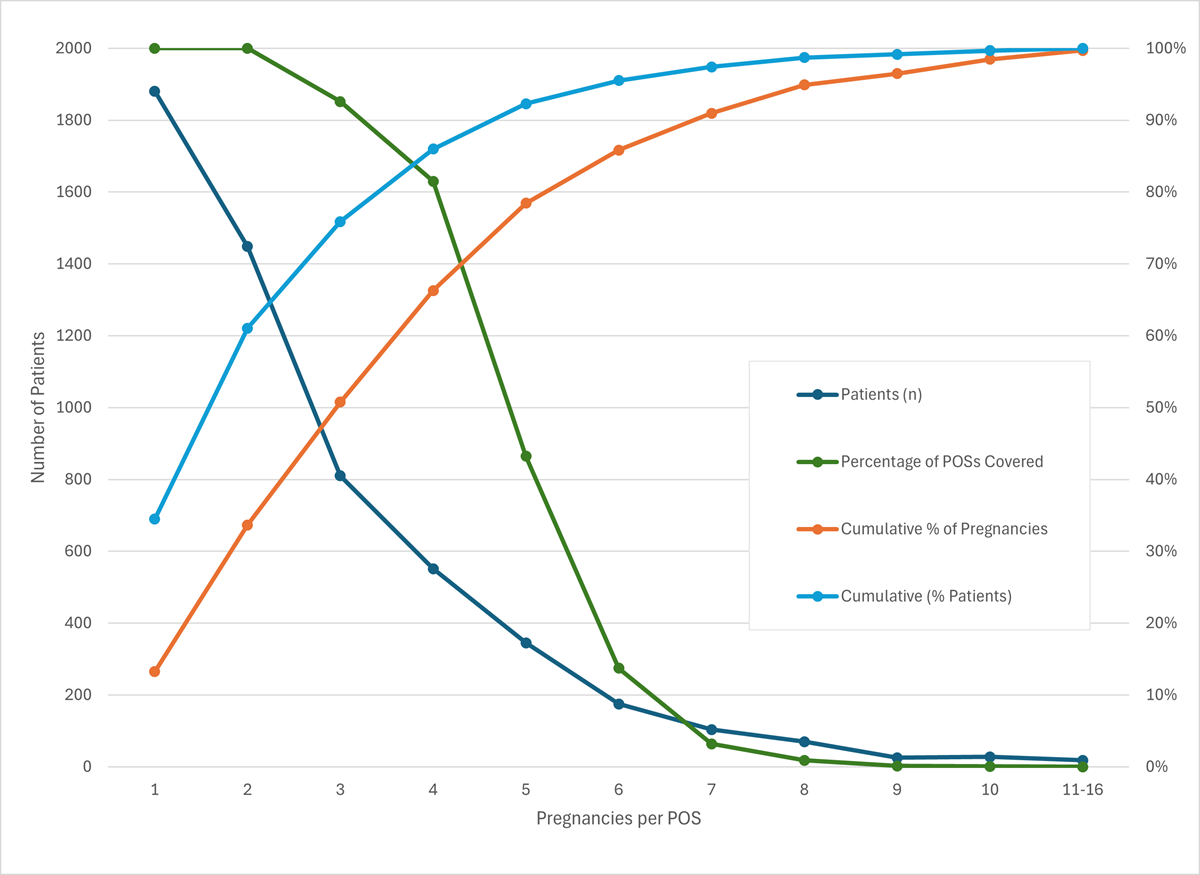
Data set POS distribution. POS: pregnancy outcome sequence.

Coding the Medicaid data using the POS structure resulted in a dataset of 5455 women and 14,198 pregnancies grouped in 508 distinct POS groups. The distribution of the number of pregnancies is highly skewed to the right, however. Table S1 in Multimedia Appendix 1 and Figure 1 show the very rapid reduction in Patients/POS with increasing numbers of pregnancies. Note that a cell size minimum of 30 women is no longer met beyond 3 pregnancies and accounts for less than 51% of all pregnancies in the data set. To account for at least 80% of all pregnancies requires including all women who have at least 6 pregnancies, with an average POS cell size of only 1.75 women. Note also that all 14,198 pregnancies populate only 508 POS groups out of a possible 64.6 million. This skewed concentration effectively precludes analyzing at the POS level the effect of sequences on pregnancy outcomes. Instead, we use an exploratory iterative sequential analytical approach to extract information from the disaggregated sequence segments through various ordering counting methods, each method addressing a different question.

#### Counting Method 1: Data Aggregation for the Correlation Matrix

The length of the POS is the number of pregnancies that a woman experiences in her lifetime. For example, suppose a woman has a total of 4 pregnancies: birth-abortion-natural loss-birth. This determines a POS of 2-1-3-2.

For each POS sequence, we created a row vector of length 4 to store the frequencies of each specific outcome and the total number of outcomes without regarding the order in which each outcome occurred. In this example, the frequency of abortion is 1, birth is 2, natural loss is 1, and the number of pregnancies is 4. Thus, the 4D row vector underlying the sequence is (1, 2, 1, 4). We compute the row vector for each sequence in the dataset and use it to compute the correlation matrix. In this application, the correlation coefficient measures the strength and direction of the association of each pair of pregnancy variables represented by the 4D row vectors. It is important to note that the unit of analysis is the count of the 4 outcomes within a given POS, disregarding their order. Therefore, the correlation coefficients are a measure of the pairwise associations between the different pregnancy variables of the POS. The coefficients are not influenced by the order, so that, for example, it does not matter whether any abortion comes before or after any natural loss. Thus, the correlation coefficients do not address the influence of the ordering of various total outcomes. Note that the statistical software R was used to obtain the correlation matrix between the 4 columns of the data set.

#### Counting Method 2: Data Aggregation for Maximum Likelihood Estimation of Specific Sequences of Pregnancy Outcomes

To produce a table of natural losses and births given the patterns of prior abortions for all of the POSs, we started with the sequences where the first pregnancy outcome is not an abortion. For each such sequence, we counted the number of births and natural losses that occur in the sequence before the first abortion occurs. If the sequence did not contain any abortion at all, we counted the number of births and natural losses in the entire sequence. We recorded the number of births and natural losses separately for each sequence. After we coded all the sequences, we aggregated the records of births and natural losses. The aggregate results of births and natural losses represent the frequency and proportions of births and natural losses given no (0) prior abortions. As an example, we compared 2 separate sequences: abortion, birth, birth, natural loss (1-2-2-3), and birth, abortion, abortion, natural loss (2-1-1-3). Thus, in the count of births and natural losses given 0 prior abortions, we skipped sequence 1-2-2-3, but we counted sequence 2-1-1-3 with a tally of one birth and no natural losses. To count the aggregate number of births and natural losses given only one prior abortion, we used the same method as that used to count births and natural losses given no (0) prior abortion. The only difference is that we started counting the births and natural losses right after the first abortion occurred. We counted until right before the second abortion occurred, if any. For instance, for sequence 1-2-2-3, the tally of births and natural losses after one prior abortion is 2 births and 1 natural loss. For sequence 2-1-1-3, the tally of births and natural losses after one (only) prior abortion is no (0) births and no (0) natural losses. For any specific (and only) prior abortion, the same method was used to count the number of natural losses and births. These counts resulted in tables that give the proportions of natural losses and births for circumstances in which there were no abortions in the POS, following any specific abortion within a POS, or in determining dose-response relationships with given numbers of prior abortions. Here, note that the estimation of the proportions obtained via this counting method is equivalent to the maximum likelihood estimation (MLE) of the proportions assuming that the POS sequences follow a multinomial distribution [23].

#### Counting Method 3: Data Aggregation for a Logistic Regression

We used logistic regression, where the dependent variable (response) is the proportion of natural losses, and the independent variables (doses) are the number of prior abortions, births, and natural losses, respectively, allowing for the detection of any dose-response relationships. A comprehensive logistic regression will include all possible effects of prior abortions, births, and natural losses, including the possible interactions between these outcomes. To populate the table for this, we counted the number of immediate births and natural losses under all the possible combinations of prior abortions, prior births, and prior natural losses. For instance, the tally of the sequence 3-2-1-1-3-1-3 is displayed in Table S2 in Multimedia Appendix 1, which should be understood as follows. The first pregnancy in the sequence 3-2-1-1-3-1-3 is 3, which is a natural loss. Prior to that natural loss, the woman had no pregnancies. Therefore, the combination (Prior Abortions, Prior Births, Prior Natural Losses) is (0, 0, 0). Consequently, the first row of the table is 0-0-0-0-1. Likewise, the second pregnancy of the sequence 3-2-1-1-3-1-3 is 2, which is a birth. Before that birth, the woman had only one pregnancy, which was a natural loss. Thus, the combination (Prior Abortions, Prior Births, Prior Natural Losses) is (0, 0, 1). Consequently, the second row of Table S2 in Multimedia Appendix 1 is 0-0-1-1-0. We skipped the third and fourth pregnancies of the sequence because these pregnancies ended in abortion. The fifth pregnancy was a 3, a natural loss. Before this natural loss, the woman had 2 abortions, one birth, and one natural loss. The combination of (Prior Abortions, Prior Births, Prior Natural Losses) is (2, 1, 1). Thus, the third row of the table is 2-1-1-0-1. We skip the sixth pregnancy since it is an abortion. The seventh pregnancy is a natural loss. Before that natural loss, the combination (Prior Abortions, Prior Births, Prior Natural Losses) is (3, 1, 2). Therefore, the fourth row of the table is 3-1-2-0-1. We used this counting method on all the sequences in the dataset and obtained an aggregation of all the results. Table S3 in Multimedia Appendix 1 is a sample of the aggregation. Note that the goal in this section is to understand the relationship between a woman’s history of pregnancy outcomes and her subsequent risk of natural loss. Thus, this counting method is a natural way of associating a woman’s pregnancy outcome with her prior history of pregnancy.

#### Counting Method 4: Data Aggregation for Simultaneous CIs

Next, we analyzed the effects of abortion, birth, and natural loss on the risk of subsequent natural loss separately, using simultaneous CIs (SCI). To do so, we got all of the row vectors from Table S3 in Multimedia Appendix 1 for which exactly 1 entry of (Prior Abortions, Prior Births, Prior Natural Losses) is different from 0. In other words, these are the rows with only prior abortions, only prior births, or only prior natural losses. We also included the row vector where all entries of (Prior Abortions, Prior Births, Prior Natural Losses) were 0 as a control. For instance, the row vector (3, 0, 0, 42, 13, 55, 0.2364) would be included because the number of prior abortions is 3, and the number of prior births and prior natural losses are 0 for each. The meaning of this row vector is that there have been 55 women whose first 3 pregnancies resulted in abortion. However, on their fourth pregnancy, out of the 55 women, 42 had a birth, and the remaining 13 had a natural loss. This corresponds to a 23.64% rate of natural loss given exactly 3 prior abortions.

All such vectors with more than 20 patients are displayed in Table S4 in Multimedia Appendix 1. This is equivalent to the MLE of the proportion of natural loss given a certain history of pregnancy.

We built SCIs for the differences in the rate of natural loss and odds ratios (ORs) underlying each level of prior abortions to determine any statistically significant difference between the different rates. Using the same methodology, we also compared the rate of natural loss and ORs underlying each level of prior births or natural losses.

We applied the Agresti et al [24] SCIs method to obtain SCIs for the differences in the proportions and the ORs between different dose levels.

#### Counting Method 5: Increasing Statistical Power of SCIs

As will be shown in the logistic regression results, the effect of prior births on subsequent natural loss is not significant. Furthermore, the SCI displayed in section 3.4 shows there is no statistically significant difference in the rate of natural loss or ORs given different levels of prior births. Note that the sample sizes for different levels of prior births used to arrive at this conclusion are reasonably large (4113, 1370, 560, 230, and 103 for 0, 1, 2, 3, and 4 prior births, respectively). Thus, we are highly confident that birth does not have a significant effect on subsequent natural loss. Therefore, we can include sequences with births that occur before the effect level we are analyzing. For instance, if we are looking at the effects of the second abortion, the sequences 1-2-1-2, 2-1-1-3, and 1-1-2 will all be included in the analysis. Therefore, we counted the outcome of the pregnancy right after the second abortion, whether it was a birth or a natural loss. For these 3 sequences given above, the tally of births after 2 abortions is 2, and the tally of natural losses is 1. The same methodology was also used when analyzing the effect of natural loss. We did so without confounding any effect, and by doing so, we increased the statistical power of the SCI and OR in detecting any statistically significant difference in the effects of prior abortions/natural losses on the rate of subsequent natural losses. The results of this counting method are displayed in Tables S5 and S6 in Multimedia Appendix 1. This counting method is equivalent to the MLE of the proportion of natural loss given a certain history of pregnancy.

## Results

### Correlation Matrix for the Dataset

**Figure 2:**
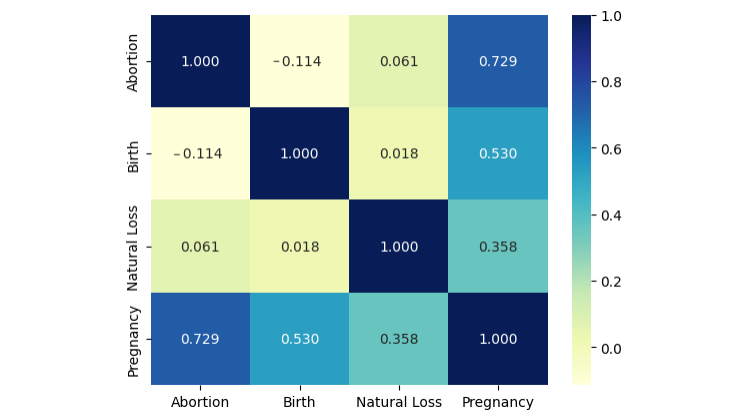
Correlation matrix.

The correlation matrix (Figure 2) shows a positive correlation between the number of pregnancies and abortions, births, and natural losses. Specifically, the correlation between the number of pregnancies and abortion is 0.73, which is the strongest among all other pairwise correlations. The correlation coefficient between pregnancy and birth is 0.53, a moderate association compared with that of pregnancy and abortion. Between pregnancy and natural loss, the correlation coefficient is 0.36, positive but relatively weak. The correlation coefficient between abortion and birth is –0.11. The correlation coefficients birth-natural loss and abortion-natural loss are respectively 0.018 and 0.061. This suggests weak associations between birth and natural loss and between abortion and natural loss at the aggregate level. Note that the results of the correlation matrix indicate that increasing pregnancies are strongly correlated to abortion, but this method is insufficiently sensitive to explain the association of prior pregnancy outcomes on the likelihood of subsequent natural loss. Therefore, we applied methods to reveal the separate marginal effect of prior pregnancies on the risk of a natural loss.

### MLE of Specific Sequences of Pregnancy Outcomes

We determined the likelihood of the outcome of the next pregnancy by identifying the pattern of pregnancy outcomes prior to it. In the simplest example, the 2030 women who had only one pregnancy outcome in their reproductive years, a birth, showed the following distribution of proportions for their next outcome: birth 55.7% (1131/2030); abortion 32.5% (660/2030); natural loss 11.8% (239/2030). For POSs that had 2 or more outcomes, the order of prior outcomes was important in determining the proportions of the third outcome. For the 359 women who had a birth first, then an abortion (2-1), the estimated proportions of the third outcome are as follows: birth 30.6% (110/359); abortion 58.2% (209/359); natural loss 11.1% (40/359). For the 289 women who had the same 2 outcomes but in the opposite order (1-2), the estimated proportions of the third outcome are: birth 45.0% (130/289); abortion 40.0% (115/289); natural loss 15.2% (44/289). Even at this 2-outcome level of analysis, the order of events was a significant determinant in the likelihood of a subsequent event.

Our research objective for this analytical phase was to determine whether there was a statistically significant difference between the proportion of natural losses for women with no prior abortions and women with at least one prior abortion. Similarly, we sought to determine the aggregate proportion of natural losses for women with 1, 2, 3, or 4 prior abortions to detect any existing dose-response association. We performed a parallel analysis for the association between prior birth and natural losses, including a no-prior-abortion requirement to isolate any effects of birth from any effects of abortion. We performed a similar analysis for the association between a prior natural loss and subsequent natural losses. Finally, we investigated the effect that the interaction of prior births, prior abortions, and prior natural losses may have on the rate of natural losses.

### Logistic Regression

Based on the aggregation result in Table S3 in Multimedia Appendix 1 we build a logistic regression model given by the following equation:



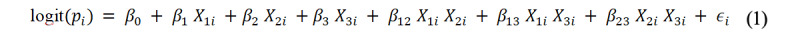



where the predictors *X*_1_*_i_*, *X*_2_*_i_*, and *X*_3_*_i_* are, respectively, the count of prior abortions, prior births, and prior miscarriages. The response, *p_i_*, is the proportion of natural loss for the pregnancy following the sequences containing exactly *X*_1_*_i_* prior abortions, *X*_2_*_i_* prior births, and *X*_3_*_i_* prior natural losses. The observations *X*_1_*_i_*, *X*_2_*_i_*, *X*_3_*_i_*, and *p_i_* are displayed in the *i*^th^ row of the aggregation result table. We only displayed a sample of the aggregation results in Table S3 in Multimedia Appendix 1 because the full table is huge. *X*_1_*_i_X*_2_*_i_*, *X*_1_*_i_X*_3_*_i_*, and *X*_2_*_i_X*_3_*_i_* are the interactions between the predictors. Furthermore, 
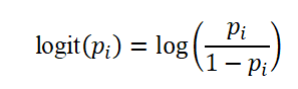
 is the log odds for the proportion of subsequent natural loss *p_i_*. Note that the goal of this model is to assess the effects and interactions of prior abortions, prior births, and prior natural losses on the risk of subsequent natural loss simultaneously. Table S7 in Multimedia Appendix 1 is the correlation matrix of the predictors. The correlation between prior abortions and prior births is 0.07, the correlation between prior abortions and prior natural losses is 0.09, and the correlation between prior births and prior natural losses is 0.15. All the correlations between the predictors are positive but weak. Thus, we detect no multicollinearity problem and retain all the predictors in our regression analysis. Table 1 displays the logistic regression results. The most significant effects are the intercept and the prior abortions, with *P* values of *<*.001. In addition, the *P* value for the effect of prior natural losses is .007 which is also very small compared with the threshold of .05. All the remaining effects and interactions (Prior births and the interactions of abortion and birth, abortion and natural loss, and birth and natural loss) have *P* values greater than .05 (*P=*.51, *P=*.60, *P=*.85, *P=*.13, respectively). We concluded that the number of prior abortions and prior natural losses has a statistically significant effect on the risk of subsequent natural loss. All the remaining effects and interactions (prior births and all the interactions) are not significant. Alternatively, we ran a reduced logistic regression model with the 2 significant effects (prior abortions and prior natural losses) while omitting all the remaining effects and interactions that are not significant. The reduced model is given by

logit(*p_i_*) = *β*_0_ + *β*_1_*X*_1_*_i_* + *β*_3_*X*_3_*_i_* + *ε_i_*. (2)

**Table 1:** Results of logistic regression for equation 1.

	Estimate	Exp(Estimate)	SE	z value	*P* value
Intercept	–1.7694	0.1704	0.0385	–45.92	*<*.001
Prior abortions	0.2085	1.2319	0.0353	5.903	<.001
Prior births	0.0213	1.0216	0.0323	0.66	.51
Prior natural loss	0.2290	1.2573	0.0862	2.656	.008
Prior abortions *×* prior births	0.0123	1.0124	0.0234	0.526	.60
Prior abortions *×* prior natural losses	–0.0086	0.9914	0.0452	–0.19	.85
Prior births *×* prior natural losses	–0.0845	0.9190	0.0557	–1.518	.13

We ran the reduced logistic regression model, and the results are displayed in Table 2. The intercept, the effects of both prior abortions and prior natural losses, are all statistically significant. Next, we determined whether the explanatory power of the reduced model (equation 2) was significantly different from that of the full model (equation 1). We performed an *F* test and the results are displayed in Table S8 in Multimedia Appendix 1.

**Table 2:** Results of logistic regression for equation 2.

	Estimate	SE	z value	*P* value
Intercept (*β*_0_)	–1.75555	0.03216	–54.586	<.001
Prior abortions (*β*_1_)	0.21849	0.02521	8.667	<.001
Prior natural losses (*β*_2_)	0.14112	0.06091	2.317	.02

The *P* value of the *F* test is .62. This is a large *P* value compared with the threshold of .05, therefore, we fail to reject the null hypothesis. Thus, the explanatory power of the full model displayed in equation 1 is quasi-equal to the explanatory power of the reduced model shown in equation 2. Note that the parsimony of the reduced model, combined with its great explanatory power, makes it a model of choice to understand the association between a woman’s history of pregnancy and her subsequent risk of natural loss. Comparing the 2 models, the intercepts of the full and reduced models are very close to each other (–1.769 and –1.7555, respectively). The coefficients of prior abortions are slightly different (0.2085 and 0.2184). The coefficient of prior natural losses looks different (0.229 vs 0.14112). Using the reduced model, the results indicate that the odds of natural loss are *e*^–1.7555^, which equals 0.17. This implies that the risk of natural loss for a woman who has no prior pregnancies is 14.5%. The coefficient underlying prior abortion is 0.2184. This implies that a unit increase in abortion changes the odds of natural loss by *e*^0.2184^, which equals 1.24. This corresponds to a 24% increase in the odds of natural loss for every additional unit of abortion, provided everything else is constant. Likewise, a unit increase in natural loss changes the odds of subsequent natural loss by *e*^0.1411^, which equals 1.15. This corresponds to a 15% increase in the odds of natural loss for every additional unit of natural loss, provided everything else is constant.

Next, we tested whether the coefficient of prior abortions, *β*_1_, in the reduced model is greater than the coefficient of prior natural losses, *β*_2_. Therefore, let *γ*=*β*_1_–*β*_0_, we solve for *β*_1_ and substitute into the logistic regression, resulting in the following equation.

logit(*p_i_*) = *β*_0_ + *γX*_1_*_i_* + *β*_2_(*X*_1_*_i_* + *X*_2_*_i_*) + *ε_i_* (3)

The results displayed in Table 3 indicate that the coefficient *γ* is not statistically significant. Therefore, we do not have significant evidence that the coefficient of prior abortions, *β*_1_, is significantly greater than the coefficient of natural losses, *β*_2_.

**Table 3:** Results of logistic regression for equation 3.

Estimate	Estimate	SE	z value	*P* value
Intercept (*β*_0_)	–1.75555	0.03216	–54.586	<.001
Difference (*γ*)	0.07737	0.06812	1.136	.26
Prior abortions + prior natural losses (*β*_2_)	0.14112	0.06091	2.317	.02

### Analysis of the SCIs

In this section, we take a different approach to analyzing the data by performing a comparative analysis of the risk of natural loss based on various categories of pregnancies. We carried out the analysis using SCIs for the difference in proportions and the ORs between the categories using 2 methods, the Agresti method (as previously described) and the Tukey-Kramer method, which tests the significance between the means for all pairs of outcomes [24,25]. Before discussing the results, we will briefly explain both methods.

Let 
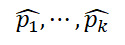
 be respectively the MLEs estimators of the proportions *p*_1_,…,*p*_k_ of *k* independent binomial populations. Define *p_ij_=p_i_* – *p_j_* and 
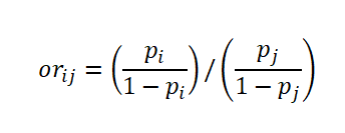
 as the difference in proportion and the OR between population *i* and population *j,* respectively, for *i*≠*j*. Clearly, the MLEs of *p_ij_* and *or_ij_* are respectively 
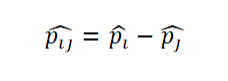
 and 
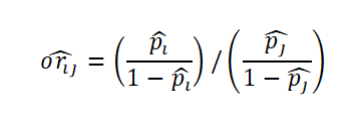
. Here, 
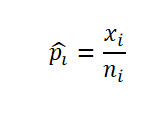
, where *x_i_* is the number of successes in a sample of *n_i_* observations from the population *i*. The 100(1 *− α*)% SCI for the pairwise differences *p_ij_* and the ORs *or_ij_* using Agresti are given by 
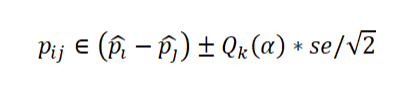
 and 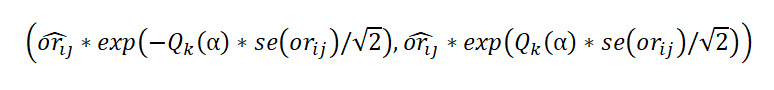 respectively, where 
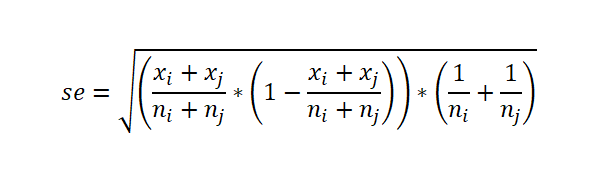
 and 
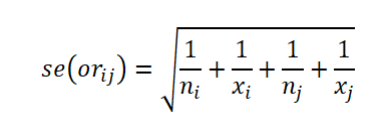


Using the Tukey-Kramer method, the 100(1*−α*)% SCI for the pairwise difference in proportion for 
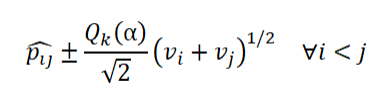
, where *Q_k_*(*α*) is the upper *α* point of the range of *k* independent and identically distributed standard normal variates. *v_i_* is the sample variance of *p*ˆ*_i_* given by 
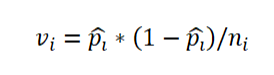
.

Next, we assessed the effect of a woman’s history of pregnancy on her subsequent risk of natural loss. We considered the data analysis from 3 perspectives. The first perspective consisted of carrying out an analysis of the effect of the number of prior abortions on the risk of natural loss. Similar to the first perspective, in the second, we analyzed the effect of the number of prior births on the risk of natural loss. In the third, we analyze the effect of the number of prior natural losses on the risk of another natural loss. In Table S9 in Multimedia Appendix 1, the aggregate numbers of births and natural losses are provided along with their corresponding proportions based on the number of prior abortions. The sample sizes (the total count of births and natural losses) underlying 0, 1, 2, 3, and 4 prior abortions are 4113, 678, 116, 55, and 33, respectively. These sample sizes are reasonably large as compared with 12, 9, 1, and 2, which are the respective sample sizes underlying 5, 6, 7, and 8 prior abortions. To avoid small sample bias, we omitted the effects of 5 or more prior abortions from our analysis. From 0 to 2 prior abortions, the proportion of natural losses tends to increase slightly (equivalently, the proportion of births tends to decrease slightly) from 14.51% (597/4113) to 15.52% (18/116). From 2 to 3 prior abortions, the proportion of natural losses increases much more, from 15.52% (18/116) to 23.64% (13/55). It then decreases from 23.64% (13/55) to 12.12% (4/33) from 3 to 4 prior abortions (Table S9 in Multimedia Appendix 1). To confirm the trend observed, we constructed 95% SCIs for the pairwise difference in proportions and the ORs using the Agresti method. For the sake of comparison, we also constructed 95% SCIs for the pairwise difference in proportions using the Tukey-Kramer method. Note that both the Agresti method and the Tukey-Kramer method for SCIs are based on the independence and normality assumptions of the sample proportions. Thus, the construction of the SCIs included those cases with 0, 1, 2, 3, and 4 prior abortions. This is because, for those cases, the sample sizes are reasonably large and we can assume that their corresponding sample proportions *p*_1_,…, *p*_4_ distributions are roughly normal by the Central Limit Theorem [26]. The 5, 6, and 7 prior abortion cases were omitted from the SCIs’ analysis due to small sample sizes.

The results of the SCIs are displayed in Tables 4 and 5. A comparison of the SCIs for the difference in proportions using the Agresti method versus the Tukey-Kramer method shows that neither method is universally superior to the other method. As shown in Tables 4 and 5, in some cases, the SCIs for the difference in proportions using the Agresti method are shorter than those of the Tukey-Kramer method and vice versa. However, all SCIs for the difference in proportions using both methods contain 0. Furthermore, all the SCIs for the ORs displayed in Table 4 contain 1. This indicates that we have no statistically significant evidence that the true proportions of natural loss *p*_0_, *p*_1_, *p*_2_, *p*_3_, and *p*_4_ differ from one another. We may interpret these results in 2 ways. The first implication could be that the number of prior abortions does not actually affect the subsequent risk of natural loss. The second implication could be that since some of the sample sizes are not that large and both tests are conservative, they could not detect any difference in the proportions of natural losses *p*_0_, *p*_1_, *p*_2_, *p*_3_, and *p*_4_. To overcome this problem, we increased the sample sizes and thereby increased the power of both tests to detect any difference in the proportions *p*_0_, *p*_1_, *p*_2_, *p*_3_, and *p*_4_, as seen in the next section.

**Table 4:** Simultaneous CIs (SCIs) for the difference in proportions and odds ratio (OR; Agresti method [24]) for the pregnancy after the ith abortion, no prior births or natural losses.

Rows	Difference in proportion	95% SCI	OR	95% SCI
(0,1)	0.005	–0.035 to 0.045	1.042923	0.762 to 1.427
(0,2)	0.010	–0.081 to 0.101	1.081735	0.536 to 2.181
(0,3)	0.091	–0.04 to 0.222	1.822924	0.779 to 4.264
(0,4)	–0.024	–0.192 to 0.144	0.8123375	0.191 to 3.46
(1,2)	0.005	–0.093 to 0.103	1.037215	0.49 to 2.194
(1,3)	0.086	–0.053 to 0.225	1.747899	0.718 to 4.255
(1,4)	–0.029	–0.202 to 0.144	0.7789047	0.179 to 3.398
(2,3)	0.081	–0.091 to 0.253	1.685185	0.567 to 5.005
(2,4)	–0.034	–0.225 to 0.157	0.7509579	0.151 to 3.723
(3,4)	–0.115	–0.352 to 0.122	0.4456233	0.084 to 2.37

**Table 5:** Simultaneous CIs (SCIs) for the difference in proportions and odds ratio (Tukey-Kramer method [25]) for the pregnancy after the ith abortion, no prior births or natural losses.

Rows	Difference in proportion	95% SCI
(0,1)	0.005	–0.035 to 0.046
(0,2)	0.010	–0.083 to 0.103
(0,3)	0.091	–0.066 to 0.248
(0,4)	–0.024	–0.18 to 0.132
(1,2)	0.005	–0.094 to 0.104
(1,3)	0.086	–0.075 to 0.247
(1,4)	–0.029	–0.189 to 0.13
(2,3)	0.081	–0.1 to 0.262
(2,4)	–0.034	–0.214 to 0.146
(3,4)	–0.115	–0.335 to 0.105

Similar to the abortion/natural loss analysis, we aggregated the count of the immediate births and natural losses based on the number of prior births, and with no prior abortion or natural loss. Here, the goal was to assess the effect of prior births on the risk of subsequent natural losses. The aggregate results displayed in Table S10 in Multimedia Appendix 1 show that the sample sizes for the total observations are respectively 4113, 1370, 560, 230, and 103 for 0, 1, 2, 3, and 4 prior births. These sample sizes are reasonably large compared with 28 and 5, which are the sample sizes for the 5 and 6 prior births. We removed the 5 and 6 prior abortion cases from our analysis. The proportions of natural losses are respectively 14.51% (597/4113), 17.45% (239/1370), 13.04% (73/560), 11.30% (26/230), and 15.53% (16/103) under 0, 1, 2, 3, or 4 prior births (Table S10 in Multimedia Appendix 1). To gain more insight from the results, we used the Agresti method and the Tukey-Kramer method to construct the 95% SCI for the difference in proportions. Note that the Agresti method also provides 95% SCI for the ORs for natural losses. As we see from Tables 6 and 7, all of the SCI for the differences in the proportion contain 0. In addition, all the SCIs for the ORs contain 1. Thus, there is no statistically significant difference between the proportions of subsequent natural losses under 0, 1, 2, 3, or 4 prior births. Here, the sample sizes used to construct the SCI are all large; therefore, we may conclude that there is no association between the number of prior births of a woman and her subsequent risk of natural loss.

**Table 6:** Simultaneous CIs (SCIs) for the difference in proportions and odds ratio (OR; Agresti method [24]) for the pregnancy after the ith birth, no prior abortions or natural losses.

Rows	Difference in proportion	95% SCI	OR	95% SCI
(0,1)	0.029	–0.001 to 0.060	1.245	0.993 to 1.559
(0,2)	–0.015	–0.058 to 0.028	0.883	0.616 to 1.265
(0,3)	–0.032	–0.097 to 0.033	0.751	0.422 to 1.336
(0,4)	0.010	–0.086 to 0.106	1.083	0.515 to 2.276
(1,2)	–0.044	–0.094 to 0.006	0.709	0.480 to 1.047
(1,3)	–0.061	–0.134 to 0.011	0.603	0.332 to 1.094
(1,4)	–0.019	–0.125 to 0.086	0.870	0.408 to 1.856
(2,3)	–0.017	–0.088 to 0.053	0.850	0.440 to 1.643
(2,4)	0.025	–0.075 to 0.125	1.227	0.547 to 2.752
(3,4)	0.042	–0.065 to 0.150	1.443	0.572 to 3.639

**Table 7:** Simultaneous CIs (SCIs) for the difference in proportions and odds ratio (Tukey-Kramer method [25]) for the pregnancy after the ith birth, no prior abortions or natural losses.

Rows	Difference in proportion	95% SCI
(0,1)	0.029	–0.002 to 0.061
(0,2)	–0.015	–0.056 to 0.027
(0,3)	–0.032	–0.091 to 0.027
(0,4)	0.01	–0.088 to 0.109
(1,2)	–0.044	–0.092 to 0.004
(1,3)	–0.061	–0.125 to 0.002
(1,4)	–0.019	–0.12 to 0.082
(2,3)	–0.017	–0.086 to 0.052
(2,4)	0.025	–0.08 to 0.13
(3,4)	0.042	–0.07 to 0.155

Similar to the first and second perspectives, here the aggregation of the count of births and natural losses is done with respect to the number of prior natural losses. Table S11 in Multimedia Appendix 1 shows that for 0 and 1 prior natural losses, the sample sizes for the total count of births and natural losses are, respectively, 4113 and 377. These sample sizes are relatively large as compared with 24 and 4, which are the sample sizes underlying 2 and 3 prior natural losses, respectively. We omitted the case with 3 prior natural losses from our analysis due to its small sample size. From 0 prior natural losses to one prior natural loss, the proportion of subsequent natural losses goes from 14.51% (597/4113) to 9.02% (34/377). From 1 to 2 prior abortions, that proportion goes from 9.02% (34/377) to 25% (6/24) (Table S11 in Multimedia Appendix 1). The constructions of the SCI using both the Agresti and the Tukey-Kramer methods show that the SCI for the difference in proportion of subsequent natural loss under 0 prior natural losses and one prior natural loss is significant. The corresponding OR is significant as well. That is, we see a significant drop in the risk of natural loss when a woman goes from 0 prior natural loss to one prior natural loss (Tables 8 and 9). However, the test could not detect any difference in the proportion of subsequent natural loss for the remaining pairs (0,2) and (1,2). This could be due to the fact that the sample size under 2 prior natural losses, which is only 24, is relatively small.

**Table 8:** Simultaneous CIs (SCIs) for the difference in proportions and odds ratio (OR; Agresti method [24]) for the pregnancy after the ith natural loss, no prior abortions or births.

Rows	Difference in proportion	95% SCI	OR	95% SCI
(0,1)	–0.055	–0.103 to –0.007	0.584	0.364 to 0.937
(0,2)	0.105	–0.081 to 0.29	1.963	0.604 to 6.376
(1,2)	0.160	–0.002 to 0.322	3.363	0.954 to 11.85

**Table 9:** Simultaneous CIs (SCIs) for the difference in proportions and odds ratio (Tukey-Kramer method [25]) for the pregnancy after the ith natural loss, no prior abortions or births.

Rows	Difference in proportion	95% SCI
(0,1)	–0.055	–0.092 to –0.018
(0,2)	0.105	–0.103 to 0.312
(1,2)	0.160	–0.05 to 0.37

The conclusion from this subsection can be summarized as follows. We found no difference in the risk of subsequent natural loss under 0, 1, 2, 3, and 4 prior births. The sample sizes used to arrive at that conclusion are large for each case (Table S10 in Multimedia Appendix 1). Therefore, we are confident to assume that there is no association between a woman’s prior history of birth and her subsequent risk of natural loss. The analysis and the statistical test performed for the risk of subsequent natural loss under prior abortions could not detect any difference in the risk of subsequent natural loss based on prior abortion history. Likewise, the statistical test could not detect any difference in the risk of subsequent natural loss for the pairs (0,2) and (1,2). In the next subsection, we performed the same analysis for prior abortions and prior natural losses. However, this time, when counting the number of prior abortions of a sequence, we allowed the prior abortions to be interspersed with some births but not with natural loss. For instance, for the following 3 sequences 1-1-2, 2-1-2-2-1-3, and 2-3-1-1-3, when we tallied the births and natural losses after 2 prior abortions, we did not take into account sequence 2-3-1-1-3 because there is a natural loss in the sequence before the 2 abortions occurred. Note that for sequences 1-1-2 and 2-1-2-2-1-3, the tally of the immediate birth and natural loss after 2 prior abortions is respectively 1 and 1. Recall, we did not find any significant effect of prior births on the risk of natural loss. Therefore, by including sequences that have a prior birth before the target number of prior abortions occurred, we are not confounding the effects of prior abortions and prior births since the effect of prior births is negligible. By using this counting method, we increased the sample sizes of the observations in our analysis and thereby increased the power of our test in detecting any statistically significant evidence about the risk of natural loss based on the number of prior abortions or the number of prior natural losses.

### Analysis of the Effect of Prior Abortions and Prior Natural Losses Under Increased Sample Sizes

By using the counting technique just described, the sample sizes of the count of immediate births and natural losses underlying 0, 1, 2, 3, and 4 prior abortions go from 4113, 678, 116, 55, and 33 to 4113, 886, 202, 109, and 53, respectively (Table S5 in Multimedia Appendix 1). Here again, we constructed 95% SCI under the increased sample sizes for the pairwise difference in proportions and the ORs for 0, 1, 2, 3, and 4 prior abortions using the Agresti method. For comparison, we also constructed 95% SCIs under the increased sample sizes for the pairwise difference in proportions using the Tukey-Kramer method. As we see from Tables 10 and 11, the Agresti method detected 4 significant effects, and the Tukey-Kramer method detected 2 significant effects. According to the Agresti method, there is a statistically significant increase in the risk of natural loss when a woman goes from 0 prior abortions to 1, 2, or 3 prior abortions. There is also a significant increase in the risk of natural loss when a woman goes from one prior abortion to 3 prior abortions. All other pairwise differences are not significant using the Agresti method. Using the Tukey-Kramer method, we found that the risk of natural loss increases significantly for women who go from 0 prior abortions to 2 or 3 prior abortions. All remaining pairwise differences are not significant. With the same counting technique, the sample sizes of the total immediate births and natural losses go from their original values of 4113, 377, and 24 to 4113, 543, and 54 under 0, 1, and 2 prior natural losses, respectively (Table S6 in Multimedia Appendix 1). To confirm these results, the Agresti and Tukey-Kramer 95% SCIs are displayed in Tables 12 and 13. The Agresti method did not detect any difference in the proportion of natural losses under 0 prior abortions and the proportion of natural losses under one prior natural loss. The Tukey-Kramer method, on the other hand, shows that the proportion of natural losses under one prior natural loss is significantly lower than that under 0 prior natural losses, suggesting that women tend to have more natural losses in their first pregnancies. Both methods show that the risk of natural loss is significantly higher for women with 2 prior natural losses as compared with women with 0 prior natural losses. Likewise, the risk of natural loss is significantly higher for women with 2 prior natural losses as compared with women with one prior natural loss. Recall that with the original sample sizes, we could not detect any difference between the proportion of natural losses between 0 and 2 prior natural losses, and also between 1 and 2 prior natural losses. Here, by increasing the sample sizes, we gain more power to detect these differences.

**Table 10:** Simultaneous CIs (SCIs) for the difference in proportions and odds ratio (OR; Agresti method [24]) for the pregnancy the ith abortion, no prior natural losses.

Rows	Difference in proportion	95% SCI	OR	95% SCI
(0,1)	0.038	0.001 to 0.074	1.318	1.014 to 1.712
(0,2)	0.092	0.022 to 0.163	1.836	1.166 to 2.89
(0,3)	0.158	0.063 to 0.252	2.557	1.468 to 4.455
(0,4)	0.100	–0.033 to 0.233	1.914	0.816 to 4.49
(1,2)	0.055	–0.029 to 0.139	1.393	0.848 to 2.288
(1,3)	0.120	0.01 to 0.23	1.941	1.076 to 3.501
(1,4)	0.062	–0.088 to 0.213	1.452	0.605 to 3.487
(2,3)	0.065	–0.077 to 0.207	1.393	0.694 to 2.797
(2,4)	0.008	–0.172 to 0.187	1.043	0.403 to 2.699
(3,4)	–0.057	–0.263 to 0.149	0.748	0.274 to 2.041

**Table 11:** Simultaneous CIs (SCIs) for the difference in proportions and odds ratio (Tukey-Kramer method [25]) for the pregnancy after the ith abortion, no prior natural losses.

Rows	Difference in proportion	95% SCI
(0,1)	0.038	–0.001 to 0.076
(0,2)	0.092	0.009 to 0.176
(0,3)	0.158	0.037 to 0.279
(0,4)	0.1	–0.062 to 0.262
(1,2)	0.055	–0.034 to 0.144
(1,3)	0.12	–0.005 to 0.245
(1,4)	0.062	–0.103 to 0.227
(2,3)	0.065	–0.08 to 0.21
(2,4)	0.008	–0.173 to 0.188
(3,4)	–0.057	–0.258 to 0.144

**Table 12:** Simultaneous CIs (SCIs) for the difference in proportions and odds ratio (OR; Agresti method [24]) for the pregnancy after the ith natural loss, no prior abortions.

Rows	Difference in proportion	95% SCI	OR	95% SCI
(0,1)	–0.038	–0.079 to 0.002	0.704	0.485 to 1.022
(0,2)	0.207	0.082 to 0.332	3.197	1.597 to 6.403
(1,2)	0.245	0.122 to 0.368	4.539	2.098 to 9.821

**Table 13:** Simultaneous CIs (SCIs) for the difference in proportions and odds ratio (Tukey-Kramer method [25]) for the pregnancy after the ith natural loss, no prior abortions.

Rows	Difference in proportion	95% SCI
(0,1)	–0.038	–0.072 to –0.005
(0,2)	0.207	0.054 to 0.36
(1,2)	0.245	0.09 to 0.4

## Discussion

### Value of Sequential Analysis

This exploratory application of sequential analyses to the formation of a reproductive history has demonstrated that the variation in the order of sequential outcomes may reveal associations not otherwise apparent using other methods. It has also revealed that sequence ordering analysis results in extraordinary complexity at scale and that the pilot application is unwieldy. Our preliminary results are internally consistent. However, limitations related to inadequate sample sizes in comparisons beyond 4 total pregnancy outcomes, exclusion of explanatory variables of likely importance, and the use of a data source selected because it enabled us to construct reproductive histories based upon certifiable, claims-based pregnancy outcomes—but without the benefit of representativeness, constrain our ability to consider the findings as confirmatory or decisive.

### Limits of Correlation Analysis

While the correlation analysis indicated that the number of total lifetime pregnancies is strongly associated with the likelihood of abortion, the correlation coefficient does not consider the ordering of outcomes within the POS nor the marginal effects of prior pregnancies on the risk of a natural loss. Therefore, it is a limited metric for determining the association of abortion with subsequent natural loss.

### Logistic Regression Findings

The logistic regression model indicated an association between the number of prior abortions or prior natural losses and an increased likelihood of subsequent natural loss. The remaining outcome (prior birth) and all interaction terms are not significant. The model indicates a 24% increase in the odds of natural loss for every additional abortion and a 15% increase for every additional natural loss, although the coefficient differences are not significant.

### SCI Analysis

An iterative analysis of the SCI for differences in the proportions and ORs using 2 separate approaches (Agresti and Tukey-Kramer methods) and increased sample size indicated that there is no association between the number of prior births and subsequent risk of natural loss; that there is evidence of a dose-response association between prior abortion and subsequent natural loss when a woman goes from 0 to 1, 2, or 3 prior abortions; and that the risk of natural loss is significantly higher for women with 2 prior natural losses and also between 1 and 2 prior natural losses. Overall, these findings suggest that a history of pregnancy loss via either induced abortion or natural loss may be associated with a significantly greater likelihood of experiencing a subsequent natural loss. The effect size on the variation is larger for prior induced abortion than for prior natural loss. For all analyses in this study, no significant association of prior births to the likelihood of natural loss is evident.

While logistic regression results suggest a dose-response association between prior abortions and subsequent natural pregnancy loss, the SCI analyses often failed to detect statistically significant differences. This inconsistency weakens the strength of the conclusions and indicates that the observed associations should be interpreted with caution.

### Study Limitations

There are serious limitations to this study. The results may not be generalizable to other populations since Medicaid eligibility is determined by financial status, which may confer other systematic biases, such as for comorbid conditions and age. Moreover, the data were restricted to Medicaid-eligible women in 7 states. Additionally, the requirement for continuous Medicaid eligibility excluded women with interrupted coverage and could have introduced selection bias if the excluded women exhibited systematically different outcome sequences than those who were continuously enrolled. Important study variables such as maternal age, comorbid conditions, and between-outcome time intervals were excluded from the parsimonious model and could risk confounding and misinterpretation of results. For example, the time intervals between repeated pregnancy outcomes are likely to be associated with the risk of any outcome in some nonlinear pattern. Complications related to consecutive births, for example, are more likely to occur if the interval between births is less than 18 months or more than 5 years. Each of the 3 basic pregnancy outcomes has a level of descriptive granularity that could result in significant variation: birth (vaginal or c-section); abortion (medication or surgical); natural loss (miscarriage, stillbirth, ectopic, and molar pregnancy). This analysis did not specify the type of induced abortion; that is, medical versus surgical, nor maternal age [20,21]. It did not distinguish between types of natural pregnancy losses, that is, miscarriage, stillbirth, ectopic pregnancies, nor neonatal losses. Without granularity, these results may mask potentially meaningful differences. To illustrate, research from a Chinese population of 2953 pregnant women concluded that women whose pregnancy was terminated by vacuum aspiration experienced an increased risk of first-trimester miscarriage in a subsequent pregnancy [9]. Lack of granularity regarding the specific type of abortion in our POS analysis limits the ability to claim consistency with these results. Similarly, recent research has concluded that in women with a history of 3 or more miscarriages, the subsequent risk may increase with the presence and number of previous caesarean births [27]. Again, lack of granularity regarding the type of birth in the POS analysis limits comparability of the findings.

The use of administrative data for claims payment is not ideal for research purposes and is subject to coding errors, the exclusion of codes considered nonessential for billing purposes, and inconsistent coding practices [28,29].

The application of the POS construct revealed other issues which could influence the analysis and the validity of these results. The dataset is highly skewed toward women with 3 or fewer pregnancies. For women with 4 or more pregnancies, the very small sample sizes produce unstable estimates and limit the robustness of the sequential outcome analysis. POSs with 3 or fewer pregnancies account for 75.9% (4139/5455) of all women and 50.8% (7207/14,198) of all pregnancies. Nearly all of the possible POSs for 3 or fewer pregnancies are represented in our sample (37 out of 39 possible). However, at 5 total pregnancies, our sample contains only 43.2% (105/243) of possible POSs, and at 8 pregnancies we capture less than 1% (60/6561) of possible POSs. As a result, for this population, we have confidence in our analysis up to 4 total pregnancies but not beyond. Cohorts of women with 4 or more pregnancies could enable valid assessment of the effects of pregnancy outcome ordering in reproductive histories with higher numbers of pregnancies. Similarly, other dimensions of the POS, aside from counts by type, are likely to affect outcomes. For example, the time intervals between outcomes and the mother’s age at each outcome are obvious candidates for inclusion.

### Future Applications of the POS Methodology

Finally, the POS may have significant utility in determining the likelihood of a range of adverse outcomes as defined by disease (eg, heart disease), health care use (eg, emergency department visits or hospital admissions), behavioral events (eg, suicidal ideation), or prescription drug use history. The seminal analysis of the POS presented here reveals the unique analytical potential of the construct but also its considerable conceptual and analytical complexity. Future development of the POS construct will address issues of scalability beyond 4 pregnancies and pursue a practical, efficient, and versatile methodology.

The POS also provides a serious cautionary note regarding research focused on a particular pregnancy outcome of interest (eg, first pregnancy and “index pregnancy”) or some count or average of all outcomes (eg, “multiple abortions”). This exploratory research suggests that the specific order of outcomes within the POS may shape the observed likelihood of subsequent outcomes. To realize the considerable research potential of the POS, investigators must have access to data which is extracted from a surveillance system in which all pregnancy outcomes are comprehensively reported. The collection of population-based health registries in Finland is a good example and widely used in medical and health services research [30]. In the United States, by contrast, valid and certified pregnancy outcome histories require the aggregation of claims payment data with all their attendant inadequacies. Nonetheless, this exploratory application of the POS demonstrates its potential capability to reveal and interpret the influence of heretofore hidden patterns of reproductive histories on various outcomes of interest. Despite this potential, substantial methodological and interpretive shortcomings identified in this pilot application significantly affect the reliability, generalizability and validity of the findings.

## Appendix

Multimedia Appendix 1 Supplementary materials.

## Abbreviations

CPT4: Current Procedural Terminology, Fourth Edition
ICD-10: International Statistical Classification of Diseases, Tenth Revision
ICD-9: International Classification of Diseases, Ninth Revision
MLE: maximum likelihood estimation
OR: odds ratio
POS: pregnancy outcome sequence
SCI: simultaneous CI

## Data Availability

The dataset analyzed during this study can be obtained through a data use agreement from the Centers for Medicare and Medicaid Services (CMS).
