# Supplementary material for: Patterns of Prior Induced Abortions and the Likelihood of Subsequent Natural Pregnancy Loss: Exploratory Application of Pregnancy Outcome Sequencing
Source: JMIR Form Res. 2025 Dec 9;9:e78489. doi: 10.2196/78489 (PMC12728397; doi:10.2196/78489)
Supplement: Multimedia Appendix 1 [file formative_v9i1e78489_app1.docx]

Table S1: POSs: total and cumulative pregnancies and patients. POS = pregnancy outcome sequence.

| **Pregnancies per Patient** | **Patients (n)** | **Patients (%)** | **Pregnancies** | **POS (n)** | **Possible POSs** | **Percentage of POSs Covered** | **Patients per POS** |
| --- | --- | --- | --- | --- | --- | --- | --- |
| 1 | 1881 | 34.48% | 1881 | 3 | 3 | 100.00% | 627.00 |
| 2 | 1448 | 26.54% | 2896 | 9 | 9 | 100.00% | 160.89 |
| 3 | 810 | 14.85% | 2430 | 25 | 27 | 92.59% | 32.40 |
| 4 | 551 | 10.10% | 2204 | 66 | 81 | 81.48% | 8.35 |
| 5 | 345 | 6.32% | 1725 | 105 | 243 | 43.21% | 3.29 |
| 6 | 175 | 3.21% | 1050 | 100 | 729 | 13.72% | 1.75 |
| 7 | 104 | 1.91% | 728 | 70 | 2187 | 3.20% | 1.49 |
| 8 | 70 | 1.28% | 560 | 60 | 6561 | 0.91% | 1.17 |
| 9 | 25 | 0.46% | 225 | 25 | 19,683 | 0.13% | 1.00 |
| 10 | 28 | 0.51% | 280 | 27 | 59,049 | 0.05% | 1.04 |
| 11-16 | 18 | 0.33% | 219 | 18 | 64,481,508 | 0.01% | 1.00 |
| **Totals** | **5455** |  | **14198** | **508** | **64,570,080** |  |  |

Table S2: Tally with the pregnancy sequence 3211313.

| **Prior Abortions** | **Prior Births** | **Prior Natural Losses** | **Births** | **Natural Losses** |
| --- | --- | --- | --- | --- |
| 0 | 0 | 0 | 0 | 1 |
| 0 | 0 | 1 | 1 | 0 |
| 2 | 1 | 1 | 0 | 1 |
| 3 | 1 | 2 | 0 | 1 |

Table S3: Table of prior outcomes (only outcomes with 30 or more observations are shown here).

| **Prior Abor- tions** | **Prior Births** | **Prior Natural Loss** | **Births** | **Natural Losses** | **Total** | **Proportion** |
| --- | --- | --- | --- | --- | --- | --- |
| 0 | 0 | 0 | 3516 | 597 | 4113 | 0.1451 |
| 0 | 0 | 1 | 343 | 34 | 377 | 0.0902 |
| 0 | 1 | 0 | 1131 | 239 | 1370 | 0.1745 |
| 0 | 1 | 1 | 172 | 38 | 210 | 0.1810 |
| 0 | 2 | 0 | 487 | 73 | 560 | 0.1304 |
| 0 | 2 | 1 | 113 | 8 | 121 | 0.0661 |
| 0 | 3 | 0 | 204 | 26 | 230 | 0.1130 |
| 0 | 3 | 1 | 51 | 5 | 56 | 0.0893 |
| 0 | 4 | 0 | 87 | 16 | 103 | 0.1553 |
| 0 | 4 | 1 | 27 | 5 | 32 | 0.1563 |
| 1 | 0 | 0 | 576 | 102 | 678 | 0.1504 |
| 1 | 0 | 1 | 85 | 12 | 97 | 0.1237 |
| 1 | 1 | 0 | 240 | 84 | 324 | 0.2593 |
| 1 | 1 | 1 | 65 | 15 | 80 | 0.1875 |
| 1 | 2 | 0 | 91 | 30 | 121 | 0.2479 |
| 1 | 2 | 1 | 33 | 6 | 39 | 0.1538 |
| 1 | 3 | 0 | 33 | 11 | 44 | 0.2500 |
| 2 | 0 | 0 | 98 | 18 | 116 | 0.1552 |
| 2 | 1 | 0 | 60 | 40 | 100 | 0.4000 |
| 3 | 0 | 0 | 42 | 13 | 55 | 0.2364 |
| 3 | 1 | 0 | 30 | 16 | 46 | 0.3478 |
| 4 | 0 | 0 | 29 | 4 | 33 | 0.1212 |

Table S4: Counting Method 4. Only showing rows where the total is greater than 20 patients.

| **Prior Abortions** | **Prior Births** | **Prior Natural Losses** | **Births** | **Natural Losses** | **Total** | **Proportion of Natural Losses** |
| --- | --- | --- | --- | --- | --- | --- |
| 0 | 0 | 0 | 3516 | 597 | 4113 | 0.1451 |
| 1 | 0 | 0 | 576 | 102 | 678 | 0.1504 |
| 2 | 0 | 0 | 98 | 18 | 116 | 0.1552 |
| 3 | 0 | 0 | 42 | 13 | 55 | 0.2364 |
| 4 | 0 | 0 | 29 | 4 | 33 | 0.1212 |
| 0 | 1 | 0 | 1131 | 239 | 1370 | 0.1745 |
| 0 | 2 | 0 | 487 | 73 | 560 | 0.1304 |
| 0 | 3 | 0 | 204 | 26 | 230 | 0.113 |
| 0 | 4 | 0 | 87 | 16 | 103 | 0.1553 |
| 0 | 5 | 0 | 23 | 5 | 28 | 0.1786 |
| 0 | 0 | 1 | 343 | 34 | 377 | 0.0902 |
| 0 | 0 | 2 | 18 | 6 | 24 | 0.25 |

Table S5: Proportions of births and natural losses for the pregnancy after the *ith* abortion, given no prior natural losses.

| **Prior Abortions** | **Births** | **Natural Losses** | **Proportion Births** | **Proportion Natural Losses** |
| --- | --- | --- | --- | --- |
| 0 | 3516 | 597 | 0.8549 | 0.1451 |
| 1 | 724 | 162 | 0.8172 | 0.1828 |
| 2 | 154 | 48 | 0.7624 | 0.2376 |
| 3 | 76 | 33 | 0.6972 | 0.3028 |
| 4 | 40 | 13 | 0.7547 | 0.2453 |

Table S6: Proportions of births and natural losses for the pregnancy after the *ith* natural loss, given no prior abortions.

| **Prior Natural Losses** | **Births** | **Natural Losses** | **Proportion Births** | **Proportion Natural Losses** |
| --- | --- | --- | --- | --- |
| 0 | 3516 | 597 | 0.8549 | 0.1451 |
| 1 | 485 | 58 | 0.8932 | 0.1068 |
| 2 | 35 | 19 | 0.6481 | 0.3519 |

Table S7: Correlation matrix for prior abortions, prior births, and prior natural loss.

|  | **Prior Abortions** | **Prior Births** | **Prior Natural Losses** |
| --- | --- | --- | --- |
| **Prior Abortions** | 1 | 0.07239582 | 0.09097688 |
| **Prior Births** | 0.07239582 | 1 | 0.14719326 |
| **Prior Natural Losses** | 0.09097688 | 0.14719326 | 1 |

Table S8: *F*-test for reduced vs. full models.

| **Resid.** | **Df** | **Resid. Dev** | **Df** | **Deviance** | **Pr(*>*Chi)** |
| --- | --- | --- | --- | --- | --- |
| 1 | 123 | 276.0995 | N/A | N/A | N/A |
| 2 | 119 | 273.4619 | 4 | 2.637535 | *P*=.62 |

Table S9: Proportions of births and natural losses for the pregnancy after the *ith* abortion, given no prior births or natural losses.

| **Prior Abortions** | **Births** | **Natural Losses** | **Proportion Births** | **Proportion Natural Losses** |
| --- | --- | --- | --- | --- |
| 0 | 3516 | 597 | 0.8549 | 0.1451 |
| 1 | 576 | 102 | 0.8496 | 0.1504 |
| 2 | 98 | 18 | 0.8448 | 0.1552 |
| 3 | 42 | 13 | 0.7636 | 0.2364 |
| 4 | 29 | 4 | 0.8788 | 0.1212 |

Table S10: Proportions of births and natural losses for the pregnancy after the *ith* birth, given no prior abortion and no prior natural loss.

| **Prior Births** | **Births** | **Natural Losses** | **Proportion Births** | **Proportions Natural Losses** |
| --- | --- | --- | --- | --- |
| 0 | 3516 | 597 | 0.8549 | 0.1451 |
| 1 | 1131 | 239 | 0.8255 | 0.1745 |
| 2 | 487 | 73 | 0.8696 | 0.1304 |
| 3 | 204 | 26 | 0.8870 | 0.1130 |
| 4 | 87 | 16 | 0.8447 | 0.1553 |

Table S11: Proportions of births and natural losses for the pregnancy after the *ith* natural loss, given no prior abortions or births.

| **Prior Natural Losses** | **Births** | **Natural Losses** | **Proportion Births** | **Proportion Natural Losses** |
| --- | --- | --- | --- | --- |
| 0 | 3516 | 597 | 0.8549 | 0.1451 |
| 1 | 343 | 34 | 0.9098 | 0.0902 |
| 2 | 18 | 6 | 0.75 | 0.25 |
